# The Strica Homolog AaCASPS16 Is Involved in Apoptosis in the Yellow Fever Vector, *Aedes albopictus*

**DOI:** 10.1371/journal.pone.0157846

**Published:** 2016-06-28

**Authors:** Kun Meng, Xiaomei Li, Shengya Wang, Chunyan Zhong, Zhouning Yang, Lingyan Feng, Qingzhen Liu

**Affiliations:** State Key Laboratory of Virology and Modern Virology Research Center, College of Life Sciences, Wuhan University, Wuhan, People's Republic of China; Chinese Academy of Sciences, CHINA

## Abstract

Caspases are a family of cysteine proteases playing essential roles during apoptosis. Seven caspases identified in *Drosophila* were Dronc, Dredd, Strica, Dcp-1, Decay, Drice and Damm. Among them, Strica is an insect-specific caspase containing a long serine- and threonine- rich prodomain, of which function is not yet well studied. Here we identified a homolog of *strica* from *Aedes albopictus*, named as *Aacasps16*. *Aacasps16* encoded a protein containing a putative serine- and threonine-rich prodomain and a well conserved caspase catalytic domain. AaCASPS16 shared high identity with dipteran insects Strica homologs. Alignment showed that the closest relative of AaCASPS16 was *Aedes aegypti* AeCASPS16. The expression profiles of *Aacasps16* during developmental and adult stages were analyzed. Purified recombinant AaCASPS16 exhibited the highest caspase activity to WEHD, which is the substrate preferred by human caspase-9. AaCASPS16 induced apoptosis when over-expressed in C6/36 cells. AaCASPS16 was processed during apoptosis induced by actinomycin D and ultraviolet irradiation treatment, whereas partial silencing of *Aacasps16* reduced actinomycin D- and ultraviolet irradiation-triggered apoptosis in C6/36 cells. Taken together, our study identified AaCASPS16 as a novel apoptotic caspase in *Aedes albopictus*.

## Introduction

*Aedes albopictus* mosquito is an efficient vector for the transmission of arboviruses, such as DENV and CHIKV. It has spread around the world rapidly, and is becoming a serious threat to public health [1, 2]. Understanding antiviral defense mechanisms in *Aedes albopictus* is critical to develop strategies to control arbovirus transmission [3].

Apoptosis, a conserved cellular process to eliminate unwanted or damaged cells, is a potential antiviral mechanism in insects. Apoptosis is triggered in salivary glands of *Culex quinquefasciatus* upon infection with West Nile virus, and this apoptosis has been proposed to be a defense against infection [4]. In addition, it has been proposed that apoptosis limits the number of WNV-infected *Culex pipiens* epithelial cells and inhibits disseminated viral infections from the mosquito midgut [5]. Also, recombinant Sindbis virus expressing *Drosophila* IAP antagonist Rpr induced apoptosis and replicated defectively in *Aedes aegypti* compared with control Sindbis virus [6]. However, the roles of apoptosis in the interaction between arboviruses and mosquito vector remain to be further elucidated.

Apoptosis is orchestrated by the concerted actions of caspases, which are a family of proteases that possess a conserved cysteine active site and cleave substrates after aspartic acid residues [7]. Caspases are originally synthesized as inactive procaspases. Upon receiving an apoptotic signal, procaspase is cleaved into a large subunit and a small subunit, and prodomain is released [8]. One large and one small subunit heterodimerizes and two heterodimers form a fully active caspase. Apoptotic caspases are divided into initiator caspase with long prodomain and effector caspase with short prodomain. After activated, initiator caspases cleave and activate effector caspases [9]. Once activated, effector caspases cleave a large number of proteins that ultimately lead to apoptosis [10].

In *Drosophila*, seven caspases have been identified. Among them, Dronc, Dredd and Strica are initiator caspases whereas Drice, Dcp-1, Damm and Decay are classified as effector caspases [11]. Although Dronc, Dredd, Strica each contain a long prodomain, they exhibit different functions and activation pathways. Dronc is proved to be essential for apoptosis [12], but Dredd seems principally related to innate immunity [13]. Strica, an insect-specific caspase, contains a long serine- and threonine- rich prodomain [14] and is assigned either to a potential initiator caspase [15] or to an effector caspase [16]. Though this remains to be elucidated, some evidence indicated that Strica was involved in apoptosis. Over-expression of Strica induces apoptosis in *Drosophila* SL2 cells, and ectopic expression of Strica under the GRM promoter in *Drosophila* results in a rough eye phenotype [14]. *In vivo* RNAi analysis reveals that Strica functions in Hid-mediated apoptosis that is partially suppressed by DIAP1 [17]. In addition, Strica was reported to function in a redundant fashion with Dronc in multiple biological processes including apoptosis during oogenesis [18], early metamorphosis in *Drosophila* [19]. Besides that, Strica is also involved in competitive apoptosis in Drosophila cells in which ribosomal protein genes are mutated [20].

In *Aedes aegypti*, four homologs (*Aecasps15*, *Aecasps16*, *Aecasps17* and *Aecasps21*) of Strica have been identified [21]. It has been shown that transcription level of *Aecasps16* increased in the refractory strain of mosquitoes fed with blood containing type-2 Dengue virus (DENV-2), indicating that this caspase may be related to viral invasion [22]. However, no specific molecular or biochemical information is available on these putative caspases until now.

In this study, a *strica* homolog from *Aedes albopictus*, named *Aacasps16*, was identified. AaCASPS16 was predicted to contain a serine- and threonine- rich prodomain. The expression profiles of *Aacasps16* during developmental and adult stages were analyzed. Purified *E*.*coli*-expressed recombinant AaCASPS16 showed the highest activity toward WEHD, which is a preferred substrate of initiator caspases. When over-expressed in C6/36 cells, AaCASPS16 induced apoptosis. The processed AaCASPS16 increased after actinomycin D (Act D) or ultraviolet irradiation (UV) treatment, and silencing of *Aacasps16* in C6/36 cells attenuated apoptosis triggered by Act D or UV treatment. Taken together, our study identified and characterized AaCASPS16 as a novel apoptotic caspase of *Aedes albopictus*.

## Materials and Methods

### Cells and mosquitoes

C6/36 cells were kindly provided from China Center for Type Culture Collection (CCTCC). Cells were reared in our laboratory and were maintained at 28°C in minimal essential medium (MEM) (Hyclone) supplemented with 10% (v/v) heat inactivated fetal bovine serum (FBS) (Gibco) in 5% CO_2_ atmosphere.

*Aedes albopictus* were kindly provided by Hubei Provincial Center for Disease Control and Prevention (HBCDC). Larvae and pupae were fed on a mixture of finely ground cat food (Nestle, Purina). *Aedes albopictus* mosquitoes were reared at 28°C and approximately 80% relative humidity with a 16:8 light:dark photoperiod.

### Identification and sequencing of *Aacasps16* cDNA

Fragments of *Aacasps16* were amplified from C6/36 cDNA by PCR using primers designed according to cDNA sequences of *Aedes aegypti Aecasps16* (VectorBase: AAEL005956). Purified PCR products were cloned into pCR-II vector (TA Cloning® Kit; Invitrogen, CA, USA) and sequenced. The obtained partial sequences of *Aacasps16* were used to design primers for rapid amplification of cDNA ends (RACE), and 5’ and 3’ RACE of *Aacasps16* were conducted using SMARTer™ RACE cDNA Amplification Kit (Clontech, CA, USA). Products from 5’ and 3’ RACE reactions were cloned into pCR-II after purification and subjected to sequencing. The sequencing results revealed a sequence containing an open reading frame (ORF) of 1191 bp flanked by a 5’ untranslated region (UTR) of 91 bp and a 3’ UTR of 268 bp. The ORF was amplified from C6/36 cDNA by PCR and cloned into pCR-II. Plasmids from 5 positive colonies were subjected to sequencing and the sequencing results confirmed the sequence information of previous sequencing of RACE products.

### Plasmids construction

pET30a-AaCASPS16-His was constructed by cloning the coding region of AaCASPS16 containing a C-terminal His-tag into the Nde I and Xho I site of pET30a vector. pIE1-AaCASPS16-Flag was constructed by cloning the coding region of AaCASPS16 containing a C-terminal Flag-tag into the BamH I and Xho I site of pIE1 vector. pIE1-AaCASPS16-C300A and pET30a-AaCASPS16-C300A were constructed by introducing a point mutation to the corresponding wild type plasmids via overlapping PCR. The sequences of all plasmids were confirmed by DNA sequencing.

### Generation of siRNA

DNA oligonucleotides used as templates for siRNA production (S2 Table) with T7 promoter 5’-TAATACGACTCACTATAG-3’ and two guanines at the 5’ end were synthesized by Sangon Co. (Shanghai). In two independent reactions, a total of 10 pmol of sense or antisense and its complementary oligonucleotides were mixed. The mixture was denatured by heating at 95°C for 2 min and then cooled down slowly to room temperature to form a double-stranded DNA template for transcription. The transcription reaction was performed in a total volume of 20 μL and incubated at 37°C according to the manufacturer’s instructions (MEGAscript® T7 Kit). Equal amounts of sense and antisense transcription products were mixed overnight at 37°C to form hybrid siRNA. A total of 10 U of S1 nuclease (TaKaRa) and 1 U of DNase I (Fermentas) were added to 1 μg of siRNA to remove the two guanines at the 5’ end of the siRNA, the residual ssRNA and the template DNA. The siRNA was purified by phenol/chloroform extraction. The integrity and the quantity of the siRNA were monitored by electrophoresis and absorbance measurement at 260 nm.

### Recombinant protein expression and purification

BL21 *E*.*coli* cells containing expression plasmids with or without the ORF for the protein of interest were grown to a concentration of A_600_ = 0.4 in LB containing 50 μg/mL kanamycin, induced by the addition of isopropyl-β-D-thiogalactopyranoside (IPTG) to a final concentration of 0.4 mmol/L and incubated at 20°C at 220 rpm for 3 h. BL21 cells were centrifuged and resuspended in 20 mmol/L imidazole solution containing 1% Triton X-100, followed by 180 cycles of sonication for 4 s with a 6-s interval between each cycle. After centrifugation at 15,000 g for 20 min at 4°C, the supernatant was collected and then applied to 500 μL of Ni-NTA high-affinity resin (Genscript) in a gravity column. The resin was washed with a concentration gradient of imidazole solution (20 mmol/L, 50 mmol/L, 80 mmol/L) and then eluted with 250 mmol/L imidazole solution. After quality analysis by Coomassie blue staining and immunoblotting, the purified protein was stored in -70°C for later use.

### Chemical and UV treatment

To induce apoptosis, C6/36 cells were treated with Act D (PureOne) at a final concentration of 1.0 μg/mL or exposed to UV of 200 μJ/cm^2^ using a UVB 500 UV cross-linker (Hoefer Scientific Instruments). Cells were harvested at 24 h after Act D or UV treatment for later analysis. Pan caspase inhibitor Z-Val-Ala-Asp-(OMe)-Fluoromethylketone (z-VAD-FMK) (Beyotime) was added to C6/36 cells at 2 h before transfection at a final concentration of 20 μmol/L. Proteasomal inhibitor MG132 (Sigma-Aldrich) was added to medium at a final concentration of 20 μmol/L at 8 h before cells were harvested.

### Plasmid and siRNA transfection

C6/36 cells were transfected using lipofectamine 2000 (Invitrogen) according to the manufacturer’s protocol. Briefly, C6/36 cells were grown to an 80% - 90% confluence before transfection. A total of 1.6 μg of plasmid DNA or 200 pmol of siRNA dissolved in 25 μL of opti-MEM (Hyclone) was incubated with 3 μL of lipofectamine dissolved in 25 μL of opti-MEM at room temperature for 30 min before the mixture was added to the cells in 12-well plates.

### RNA preparation and cDNA synthesis

Total RNA from C6/36 cells or mosquitoes were isolated using TRIzol reagent (Invitrogen) as described by the manufacturer. Briefly, after homogenizing the sample with TRIzol, chloroform was added and homogenate is separated into three layers after centrifugation. RNA in the upper aqueous layer was precipitated with isopropanol. The precipitated RNA was washed with 75% ethanol to remove impurities and dissolved in RNase-free water. Mosquito samples were first washed by RNase-free water three times. After homogenizing in TRIzol reagent by grinding, mosquito samples were centrifuged at 12,000 g for 10 min at 4°C to remove insoluble material and subjected to RNA preparation as described above. After removal of genomic DNA by DNase I (Fermentas), 1 μg of total RNA was used in the first strand cDNA synthesis by using M-MLV Reverse Transcriptase (Invitrogen) as described by Invitrogen.

### Quantitative real-time PCR (qRT-PCR)

Quantitative real-time PCR was performed according to the description of Fast Start universal SYBR master (ROX) (Roche) protocol. PCR programs were performed as follows: 95°C for 5 min, 40 cycles of 95°C for 15 s and 60°C for 40 s with an additional dissociation stage of 1 cycle of 95°C for 15 s, 60°C for 60 s, 95°C for 15 s and 60°C for 15 s. Transcript levels of the targeted genes was normalized using the control gene *Aedes albopictus ribosomal protein s7* (*Aas7*, GenBank: JN132168.1). The gene-specific primers are list in S3 Table. Quantity values were generated using the 2^-ΔΔCt^ method as described previously [23].

### Cell lysate preparation

C6/36 cells were collected and suspended in lysis buffer (200 mmol/L Tris-HCl pH 7.4, 150 mmol/L NaCl, 1 mmol/L EDTA, 1% Triton X-100) containing a Complete Mini EDTA-free protease inhibitor cocktail tablet (Roche). After 3 freeze-thaw cycles, cells were centrifuged at 15,000 g for 10 min at 4°C, and supernatants were taken as cell lysates and stored at -80°C for further analysis. For C6/36 apoptotic cells, cells were collected first, and the supernatant was then used to harvest apoptotic bodies by centrifugation at 15,000 g for 15 min at 4°C. Collected cells and apoptotic bodies were combined and cell lysates were prepared as above.

### Caspase assay

The substrate preference of purified recombinant protein was assayed using 12 different synthetic fluorogenic caspase substrates, namely, Ac-DEVD-AFC, Ac-VDVAD-AFC, Ac-AEVD-AFC, Ac-LEHD-AFC, Ac-DMQD-AFC, Ac-WEHD-AFC, Ac-VEID-AFC, Ac-YVAD-AFC, Ac-IETD-AFC, Ac-LETD-AFC, Ac-IEPD-AFC, and Ac-LEED-AFC (MP). The activities of prepared cell lysates toward Ac-DEVD-AFC were also assayed. Briefly, purified proteins or cell lysates were mixed with Na-Citrate buffer (50 mmol/L Tris-HCl, pH 7.4, 1 mol/L Na-Citrate, 10 mmol/L DTT, 0.05% CHAPS) and 20 μmol/L of each fluorogenic substrates, and the caspase activities of each mixture were assessed after incubation at 37°C for 30 min. Fluorescence (excitation 405 nm, emission 510 nm) was measured at 37°C every 2 min for 2 h. Data obtained were used to calculate the maximum slope of each curve, and Prism 6 was used to generate the graph.

### SDS-PAGE and immunoblotting

Purified recombinant proteins or prepared cell lysates were mixed with 5 × SDS loading buffer, boiled at 100°C for 5 min, and then subjected to SDS-PAGE. Proteins were transferred to nitrocellulose membranes (GE) and subjected to the following steps. Membranes were blocked overnight by 5% nonfat milk (Sangon) in TBST and then incubated with primary antibody for 1 h at room temperature. After three washes with TBST, membranes were incubated with the HRP-conjugated second antibody (Thermo) for 30 min. After another three washes, membranes were incubated in Super Signal West Pico chemiluminescent substrate (Millipore) for 1 min, and the antibody-bound protein was detected using LAS 4000 (Fujifilm). Mouse primary antibodies against His, Flag, β-actin, and β-tubulin (Proteintech) were diluted in 1: 5000 when used in immunoblotting. The polyclonal rabbit antibody against AaCASPS16 was prepared in our laboratory and was diluted in 1: 1000 when used in immunoblotting.

## Results

### Sequence analysis of AaCASPS16

**Fig 1:**
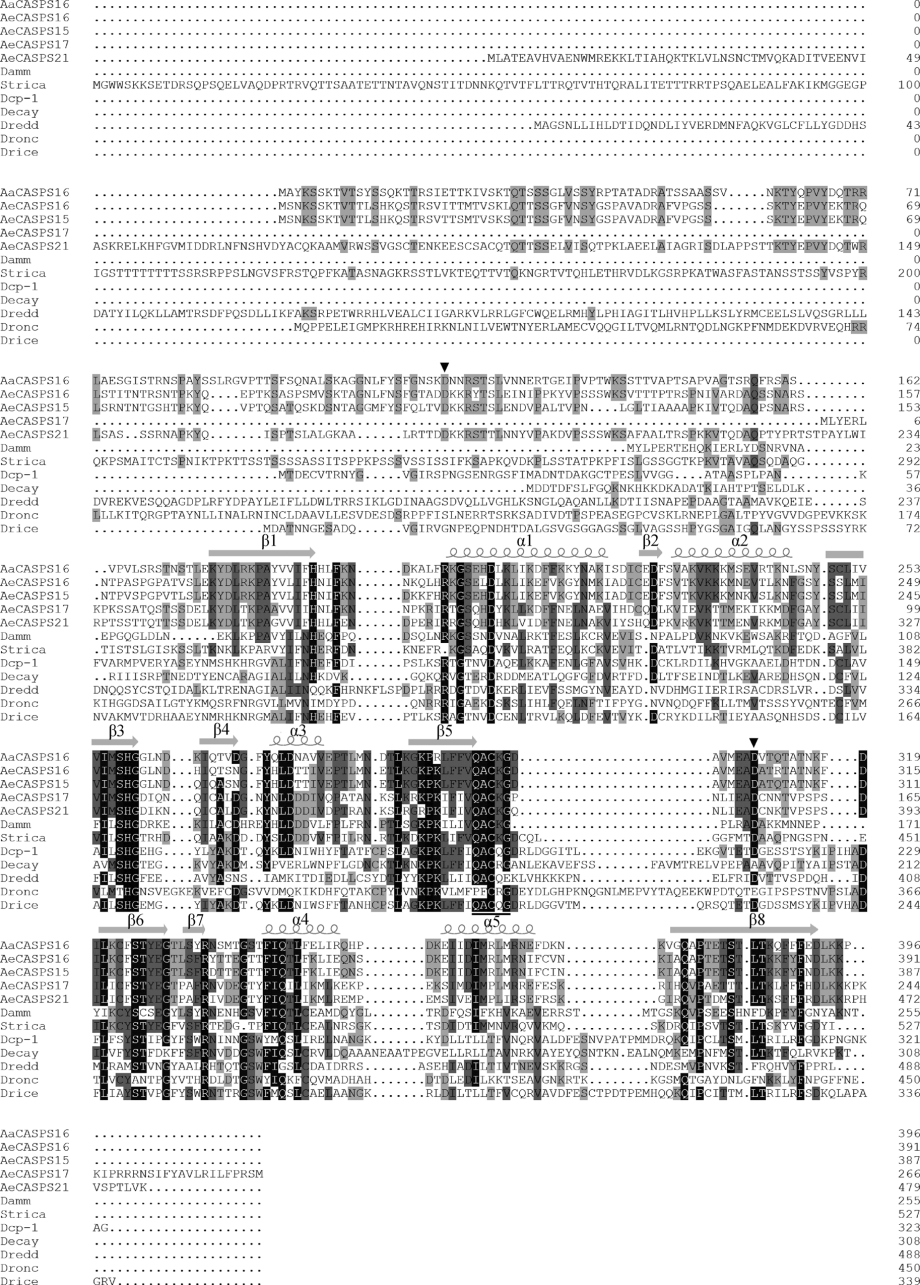
The sequence of AaCASPS16. Predicted amino acid sequence of AaCASPS16 was shown in alignment with Strica homologs from *Aedes aegypti* (AeCASPS15, AeCASPS16, AeCASPS17 and AeCASPS21) and the caspases from *Drosophila melanogaster* (Dronc, Dredd, Drice, Decay, Dcp-1, Strica and Damm). The amino acid residues identical among 12 caspases are indicated by white letters within black boxes, the amino acid residues identical among 9 caspases are indicated by black letters within dark gray boxes, the amino acid residues identical among 6 caspases are indicated by black letters within medium gray boxes, and the amino acid residues identical among 3 caspases are indicated by black letters within light gray boxes. The alignment was performed using DNAMAN 7.0. Secondary structures were predicted using JPred3. Underline: catalytic center, black arrow: predicted cleavage site.

Using primers designed according to *Aecasps16* (VectorBase: AAEL005956) in *Aedes aegypti* and cDNA of *Aedes albopictus* as template, a partial sequence of *Aacasps16* was obtained by PCR. Based on that sequence information, a fragment containing a complete ORF of 1191 bp as well as a 5’UTR of 91 bp and a 3’UTR of 268 bp was obtained by 5’ and 3’ RACE reactions. The obtained ORF sequence encoded a putative protein of 396 amino acids (predicted molecular mass of 44 kDa) with a prodomain of 12 kDa, a large subunit of 22 kDa and a small subunit of 10 kDa. The protein shared the highest similarity with *Aedes aegypti* AeCASPS16 and was therefore named AaCASPS16. Alignment of the amino acid sequences revealed that AaCASPS16 shared 62%, 60%, 50%, 44%, 39% and 33% amino acid sequence identity with AeCASPS16 (VectorBase: AAEL005956), AeCASPS15 (VectorBase: AAEL005963), AeCASPS21 (VectorBase: AAEL017498), AeCASPS17 (VectorBase: AAEL005955) of *Aedes aegypti*, Strica (GeneBank: AAF78902) and Damm (GeneBank: EDW90874) of *Drosophila melanogaster*, respectively (Fig 1). Putative AaCASPS16 possessed the typical caspase catalytic site sequence QACKG with a catalytic cysteine at position 300 (Fig 1). The predicted secondary structure of AaCASPS16 consisted of a series of alpha helices and beta sheets that were highly conserved in other caspases (Fig 1). Two possible cleavage sites were predicted according to the alignment and cleavage sites in other caspases. The predicted cleavage sites included a cleavage site between the prodomain and large subunit at D117, and a cleavage site between the large subunit and small subunit at D309 (Fig 1).

**Fig 2:**
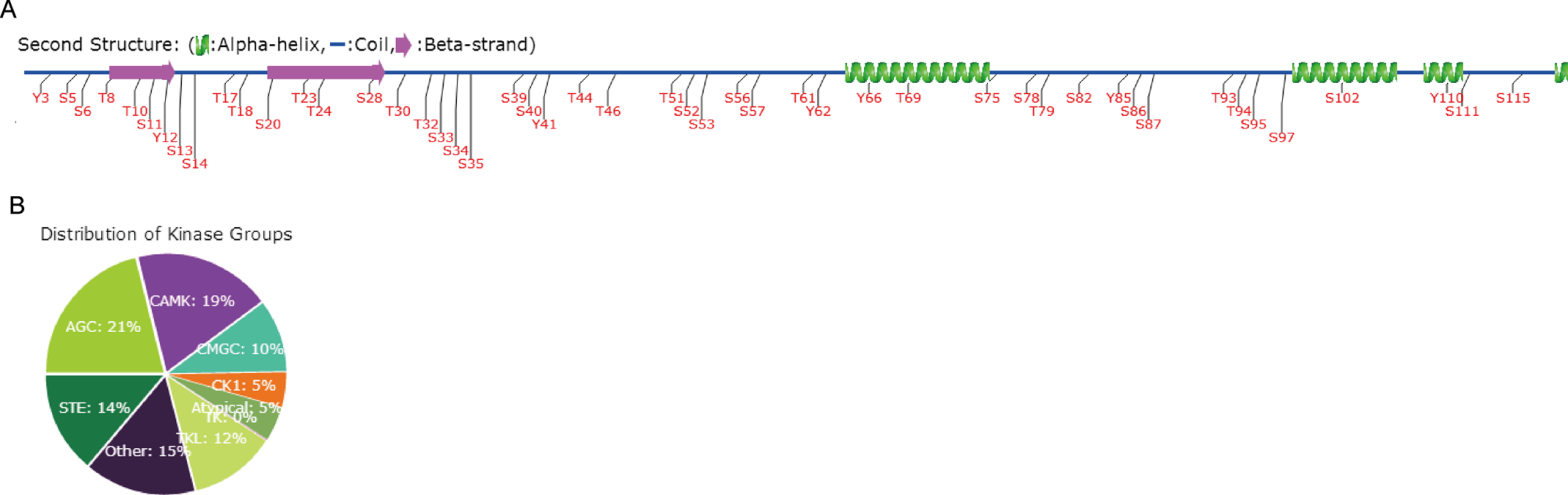
Phosphorylation sites prediction in the prodomain of AaCASPS16. Phosphorylation sites in the AaCASPS16 prodomain were predicted at the GPS web server using the default parameters [25]. (A) The numbers and positions of the potential phosphorylation sites were indicated. (B) Distribution of different potential kinase groups was calculated.

Phosphorylation of caspases is reported as a regulatory mechanism during apoptosis [24]. The serine- and threonine-rich prodomain is easily associated with phosphorylation. Unfortunately, no phosphorylation sites whthin Strica and its homologs, as well as the potential kinases were identified in regulating apoptosis pathway until now. To predict the phosphorylation sites in the prodomain of AaCASPS16, comprehensive prediction was conducted using the available database in Group-based Prediction System (GPS) system. 49 potential phosphorylation sites, including 26 serines, 16 threonines and 7 tyrosines were predicted in the 117-amino acid-long prodomain (Fig 2A). Most of these sites were predicted as substrates of serine/threonine kinases: 21% of the sites are predicted to be phosphorylated by AGC kinases group (e.g., AKT, PDK1) and 19% of the sites are predicted to be phosphorylated by CAMK kinases group (e.g., CAMKI, CAMKII) (Fig 2B).

### Phylogenetic analyses of AaCASPS16

**Fig 3:**
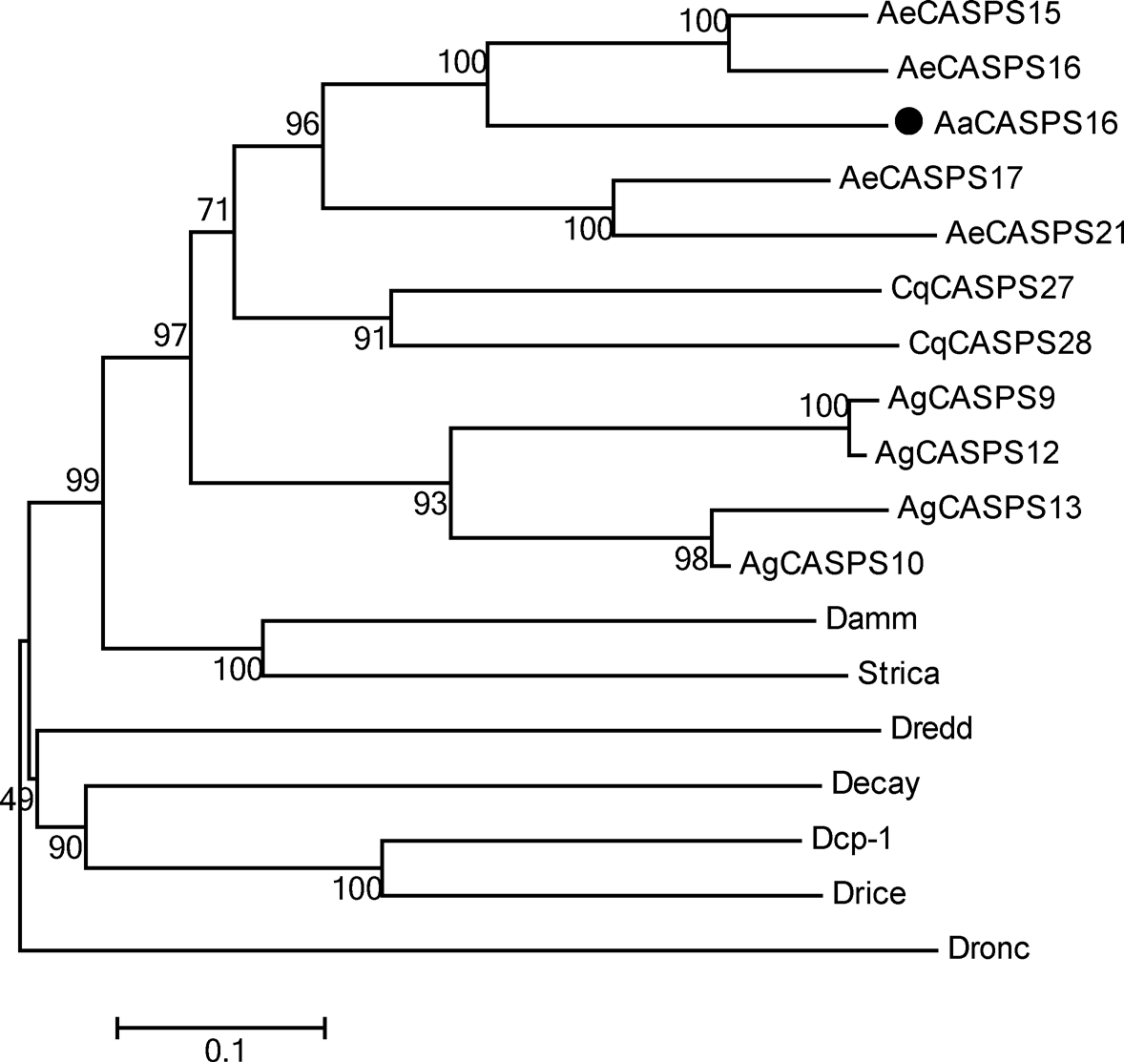
Phylogenetic analyses of AaCASPS16 with selected insect caspases. The predicted amino acid sequence of AaCASPS16 was aligned with 17 selected insect caspases, and a phylogenetic tree was constructed in MEGA 5.0 using the neighbor-joining method. AaCASPS16 was indicated by black dot. Accession numbers in GenBank and VectorBase of sequences are provided in S1 Table.

BLAST analyses against available sequences in VectorBase indicated that Strica homologs also existed in other mosquito species, including *Aedes aegypti* (AeCASPS15, AeCASPS16, AeCASPS17 and AeCASPS21), *Anopheles gambiae* (AgCASPS9, AgCASPS10, AgCASPS12 and AgCASPS13) and *Clex qinguefasiatus* (CqCASPS27 and CqCASPS28). Phylogenetic analyses of AaCASPS16 together with 17 selected insect caspases indicated that AaCASPS16 belonged to the clade of Strica homologs and that the closest relatives of AaCASPS16 were AeCASPS15 and AeCASPS16 in *Aedes aegypti *(Fig 3).

### *Aacasps16* expression profile in developmental and adult stages

**Fig 4:**
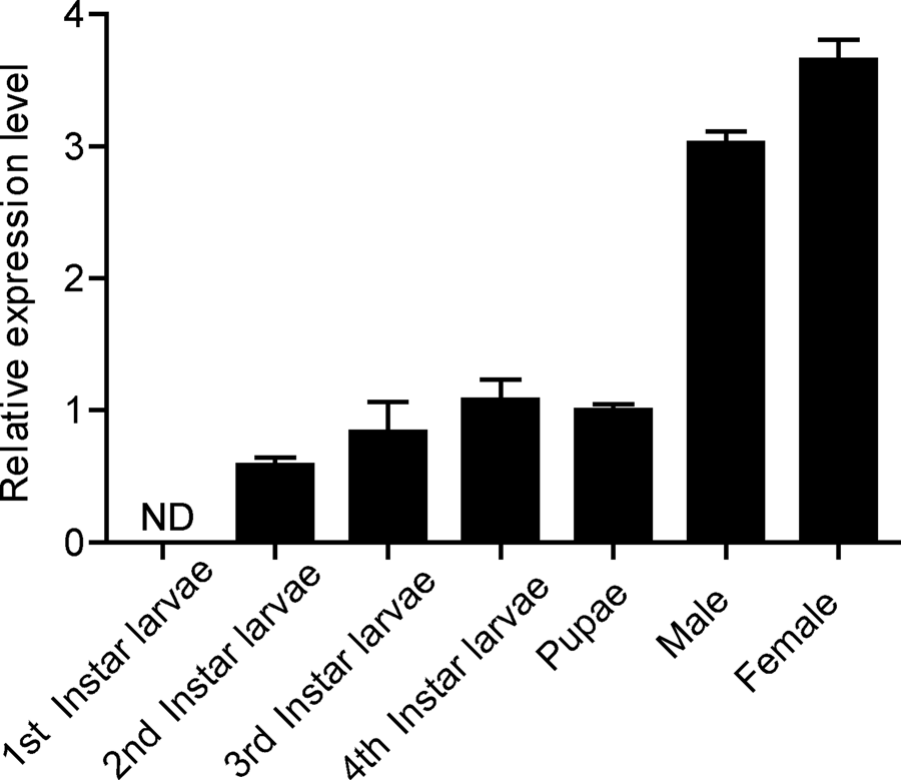
Expression profile of *Aacasps16* in developmental and adult stages. Total RNAs were prepared from 1st to 4th instar larvae, pupae, female and male adults and subjected to qRT-PCR analysis. The vertical axis represents the relative expression of *Aacasps16* in different developmental stages or different genders. Pupae samples were designated as the standard and set to 1. The data were presented with the SD from three independent experiments. ND: not detected.

To analyze the expression profile of *Aacasps16* in *Aedes albopictus* mosquito, qRT-PCR was used to quantify the transcriptional levels of *Aacasps16* in 1st to 4th instar larvae, pupae and adult mosquitoes of *Aedes albopictus*. *Aedes albopictus ribosomal protein s7* (*Aas7*, GenBank: JN132168.1) was used for normalization. *Aacasps16* was not detected in 1st instar larvae but was ubiquitously expressed in other development stages. *Aacasps16* was present at similar levels in the 2nd, 3rd, and 4th instar larvae and the pupae. Transcript levels were higher in adults than in other stages (Fig 4), which was similar to that of *strica* homologs in *Aedes aegypti*. As it is reported that the transcriptional expression of *strica* homologs (*Aecasps16*, *Aecasps17* and *Aecasps21*) was much higher in adult than in other stages including larvae of all instars and pupae [21]. Also, higher level of *Aacasps16* in female adult compared to male adult was observed (Fig 4).

### AaCASPS16 underwent autocatalytic cleavage when expressed from *E*. *coli*

**Fig 5:**
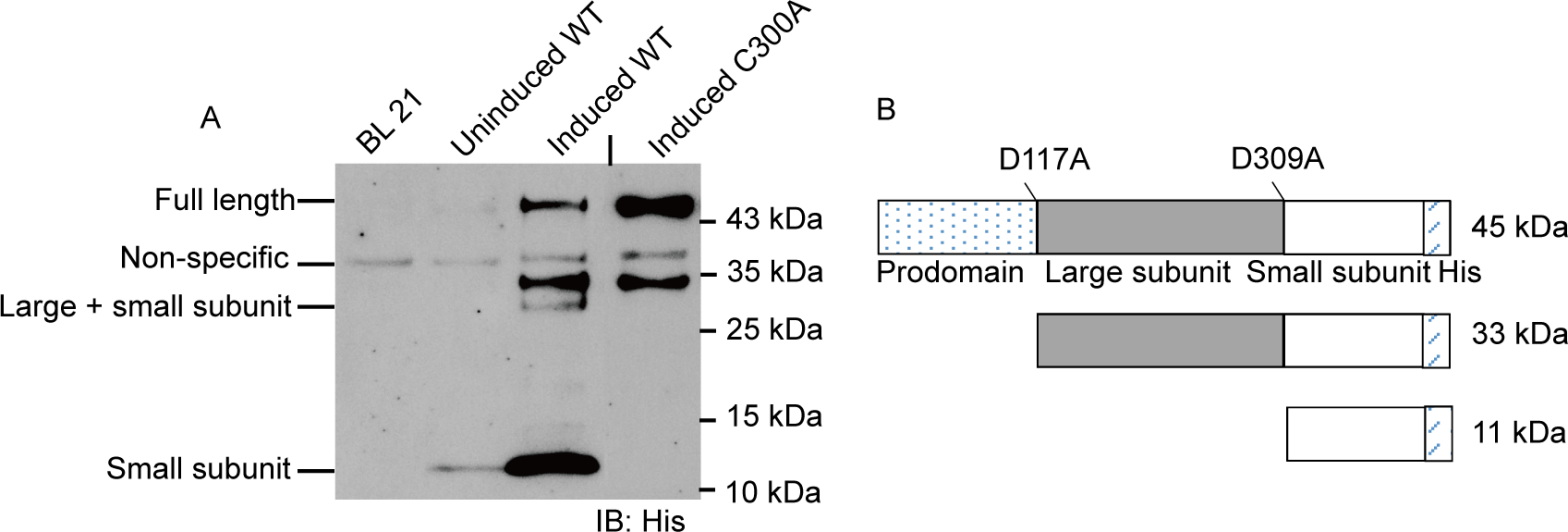
AaCASPS16 underwent autocatalytic cleavage when expressed from *E*. *coli*. (A) C-terminally His-tagged AaCASPS16-WT (lane 3) and the putative catalytic site mutant C300A (lane 4) were expressed from *E*. *coli* and detected by immunoblotting using an antibody against the His-tag following SDS-PAGE. The migration of full-length AaCASPS16 and the cleaved subunits were indicated. BL21 only (lane 1) and the uninduced WT preparations (lane 2) were used as control. A short vertical black line was used to indicate where lanes were removed and separate parts of the same Western blot image were joined together. (B) The schematic cartoon showing the molecular masses of the His-tagged full length AaCASPS16 with potential cleavage sites and the cleaved subunits.

According to the “induced-proximity” model, caspases often autoprocess themselves when brought close to each other [26]. Consistent with this, three bands with molecular masses of approximately 45 kDa, 33 kDa, and 11 kDa were observed when C-terminally His-tagged AaCASPS16 was expressed from *E*. *coli* and monitored by immunoblotting. The 45-kDa band matched the C-terminal His-tagged full-length recombinant AaCASPS16. The 33-kDa band matched the predicted fragment containing the large subunit plus the small subunit with the C-terminal His tag. The 11-kDa band matched the predicted small subunit containing the C-terminal His tag (Fig 5A lane 2 and Fig 5B). These cleaved bands suggested that AaCASPS16 underwent autocatalytic cleavage when expressed in *E*. *coli*. To study whether the observed autocleavage of AaCASPS16 was caspase activity dependent, the cysteine at position 300 (C300) in the predicted active site of AaCASPS16 was mutated to alanine. Mutant protein AaCASPS16-C300A was expressed from *E*. *coli* and subjected to immunoblotting analysis. Unlike the wild type AaCASPS16 (WT), active site mutant did not undergo autocleavage, indicated by the fact that only one major band matching the full length AaCASPS16 was observed when C-terminally His-tagged AaCASPS16-C300A mutant protein was expressed from *E*.*coli* (Fig 5A lane 1). Notably, an approximate 34-kDa band observed in AaCASPS16 WT and AaCASPS16 C300A was absent in other lanes. We speculated that AaCASPS16 was susceptible to hydrolysis or cleavage by *E*.*coli* peptidases to generate the smaller protein, as proteolytic degradation is commonly observed upon heterologous protein over expression in *E*.*coli* [27, 28]. The above data indicated that the caspase activity was required for the observed autocatalytic cleavage of AaCASPS16.

### AaCASPS16 possessed the strongest activity on substrates of initiator caspase

**Fig 6:**
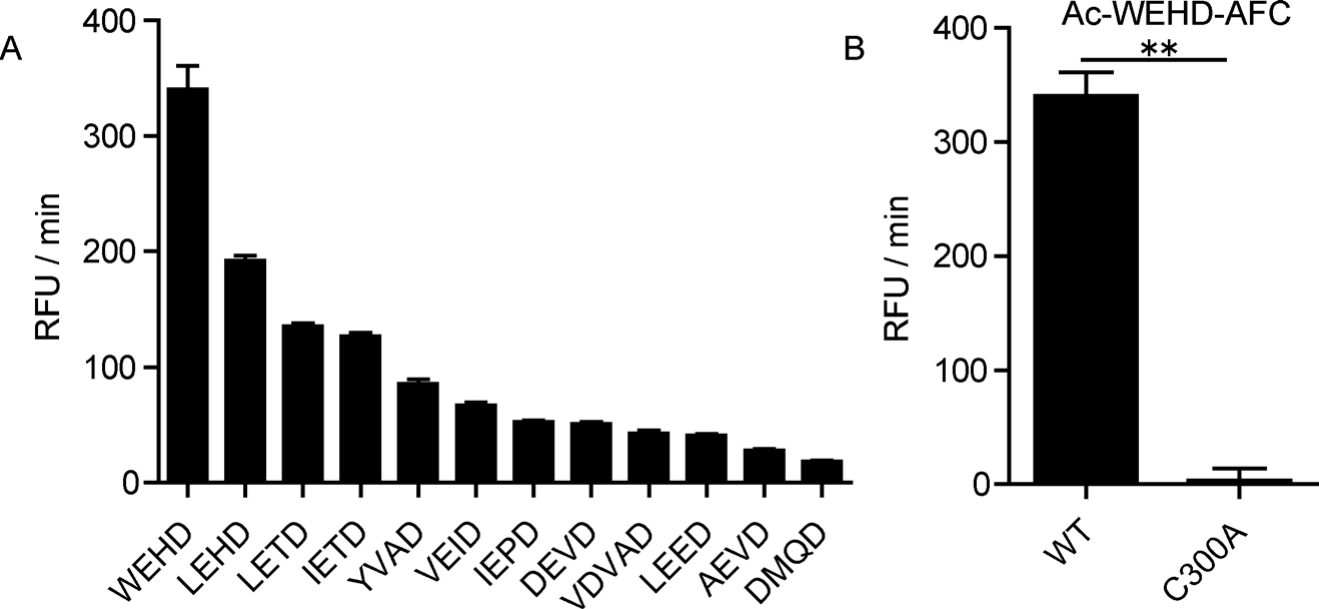
Recombinant AaCASPS16 possessed the strongest activity on initiator caspase substrates. **(A)** Substrate specificity of purified AaCASPS16 was examined against 12 different fluorescence synthetic caspase substrates. AaCASPS16 expressed and purified from *E*.*coli* (50 nM) was incubated with each substrate for 30 min, and caspase activity was indicated as the changes in relative fluorescence units (RFU) per minute. **(B)** The enzymatic activity of AaCASPS16 and the catalytic site mutant C300A were assayed by incubating with Ac-WEHD-AFC and monitoring substrate cleavage. The data were presented with the SD from three independent experiments, and statistical significance was calculated by *t* test, **P< 0.01.

Synthetic caspase substrates are widely used in detecting caspase activity [29]. To confirm that AaCASPS16 indeed possessed caspase activity, enzymatic activities of C-terminally His-tagged AaCASPS16 recombinant protein against 12 different types of synthetic caspase substrates were assayed. AaCASPS16 showed high enzymatic activity toward the substrates of human initiator caspases, including Ac-WEHD-AFC, Ac-LEHD-AFC, Ac-LETD-AFC, and Ac-IETD-AFC. Relatively low caspase activity was observed against effector caspases substrates Ac-DEVD-AFC, Ac-LEED-AMC, Ac-AEVD-AFC and Ac-DMQD-AFC (Fig 6A). C-terminally His-tagged active site mutant AaCASPS16-C300A showed no activity on the optimal substrate Ac-WEHD-AFC. The fact that the caspase active site mutation led to a complete loss of AaCASPS16 activity indicated that the activity detected for the wild type AaCASPS16 was conferred by its caspase activity (Fig 6B). The above data proved that *E*. *coli*-expressed recombinant AaCASPS16 possessed the strongest activity on initiator caspase substrates.

### Transient expression of AaCASPS16 induced apoptosis in C6/36 cells

**Fig 7:**
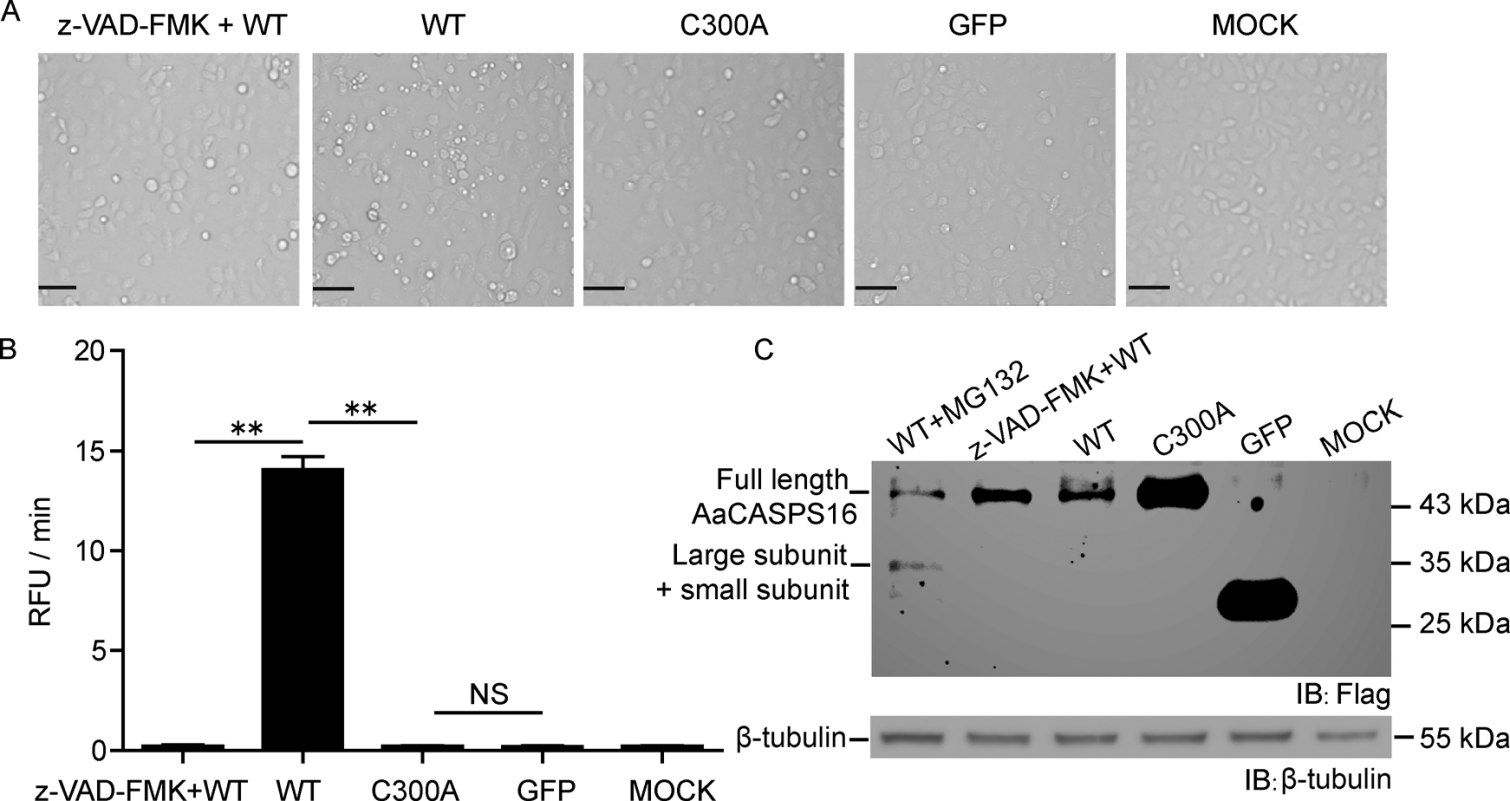
Transient expression of AaCASPS16 induced apoptosis in C6/36 cells. Plasmids expressing C-terminally Flag-tagged AaCASPS16-WT, AaCASPS16-C300A and GFP were transfected into C6/36 cells separately. Caspase inhibitor z-VAD-FMK was added at 2 h before transfection of pIE-AaCASPS16, and proteasome inhibitor MG132 was added at 8 h before cells were harvested. Mock treated cells, GFP expressed C6/36 cells, and AaCASPS16-C300A expressed C6/36 cells were used as controls. At 24 h post transfection, cells were subjected to the following analyses: **(A)** Photographs of cells were taken under microscope (Scale bar indicated 50 μm). **(B)** Cell lysates were prepared and were incubated with Ac-DEVD-AFC and subjected to caspase activity assay. Caspase activity was indicated as the changes in relative fluorescence units (RFU) per minute. **(C)** Cell lysates were subjected to immunoblotting analysis using antibody against Flag and β-tubulin. The data in (B) were presented with the SD from three independent experiments, and statistical significance was calculated by *t* test, ** *P* < 0.01. NS: not significant.

To investigate whether AaCASPS16 plays a role in apoptosis in mosquito cells, C6/36 cells were transfected with plasmids expressing C-terminally Flag-tagged AaCASPS16, active site mutant AaCASPS16-C300A and a control plasmid expressing Flag-tagged GFP. At 24 h post transfection, AaCASPS16-expressed C6/36 cells exhibited obvious apoptosis, whereas cells transfected with plasmids expressing AaCASPS16-C300A or GFP appeared normal under the microscope. The observed apoptosis induced by AaCASPS16 was attenuated by the addition of the caspase inhibitor z-VAD-FMK prior to transfection of plasmid expressing AaCASPS16 (Fig 7A), indicating that apoptosis induced by AaCASPS16 was caspase activity dependent. Consistent with these results, caspase activity to effector caspase substrate Ac-DEVD-AFC was significantly higher in C6/36 cells transfected by AaCASPS16 comparing to other groups (Fig 7B).

To further investigate the apoptosis induced by transient expression of AaCASPS16 in C6/36 cells, cells were harvested and subjected to immunoblotting analysis. Notably, the full-length band of AaCASPS16 increased after z-VAD-FMK was added (Fig 7C lane 2 and lane 3), indicating that this inhibitor may block the cleavage of AaCASPS16, thus preventing cells from undergoing AaCASPS16-triggered apoptosis. However, the band for the cleaved large plus small subunit was detectable only when the proteasome inhibitor MG132 was added, indicating that the processed band was unstable due to degradation in apoptosis (Fig 7C lane 1).

### AaCASPS16 was involved in apoptosis triggered by UV and Act D treatment in C6/36 cells

**Fig 8:**
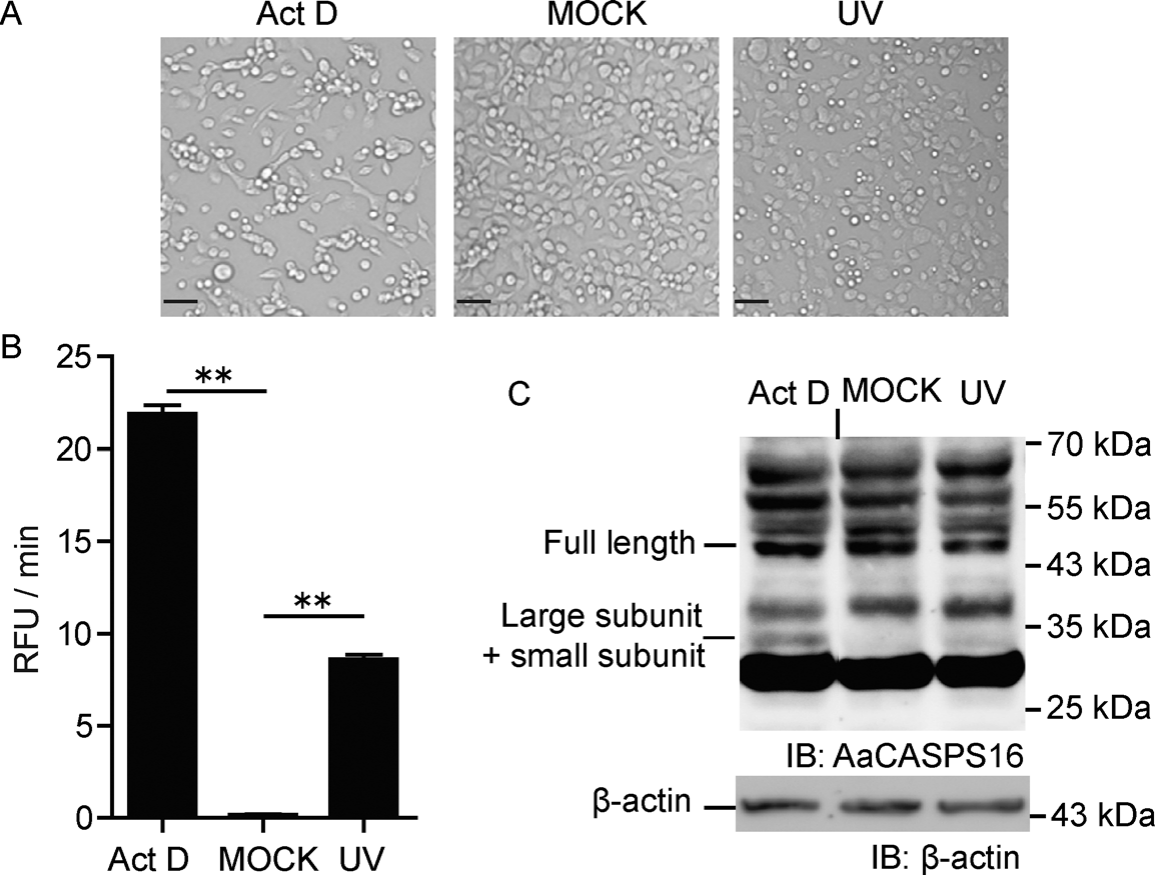
AaCASPS16 was processed in apoptosis triggered by UV and Act D treatment. C6/36 cells were treated with Act D (1.0 μg/mL) or UV treatment (200 μJ/cm^2^) separately and at 24 h post treatment, cells were subjected to the following analyses: **(A)** Cell pictures were taken under microscope (Scale bar indicated 50 μm). **(B)** Cell lysates were prepared and incubated with Ac-DEVD-AFC and subjected to caspase activity assay. Caspase activity was indicated as the changes in relative fluorescence units (RFU) per minute. **(C)** Cell lysates were analyzed by immunoblotting using antibody against AaCASPS16 and β-actin. A short vertical black line was used to indicate where lanes were removed and separate parts of the same Western blot image were joined together. The data in (B) were presented with the SD from three independent experiments, and statistical significance was calculated by *t* test, **P < 0.01.

To study the function of AaCASPS16 in the apoptosis pathway in C6/36 cells, C6/36 cells were treated with apoptosis stimulus Act D and UV, and 24 h later, we examined the cells’ morphology, measured the caspase activity and evaluated the processing level of AaCASPS16 using immunoblotting analysis. Apoptotic bodies were produced after Act D or UV treatment, thus indicating apoptosis of C6/36 cells (Fig 8A). Consistent with the morphological changes, caspase activity against the effector caspase substrate Ac-DEVD-AFC was much higher (Fig 8B). Immunoblotting with an antibody against AaCASPS16 indicated the processing of endogenous AaCASPS16. A processed product of 33 kDa, corresponding to the large subunit plus small subunit, consistent with the processed band in the recombinant His-tagged AaCASPS16 (Fig 5), was increased in apoptotic cells (Fig 8C). Notably, the fact that more processed AaCASPS16 existed in the Act D-treated cells than that in UV-treated cells, was consistent with the fact that Act D-triggered apoptosis was more obvious than UV-triggered apoptosis, indicating that the processing of AaCASPS16 was apoptosis-dependent. The processing of caspases is critical for their activation; hence, our results suggested that AaCASPS16 was activated during the apoptosis cascade.

**Fig 9:**
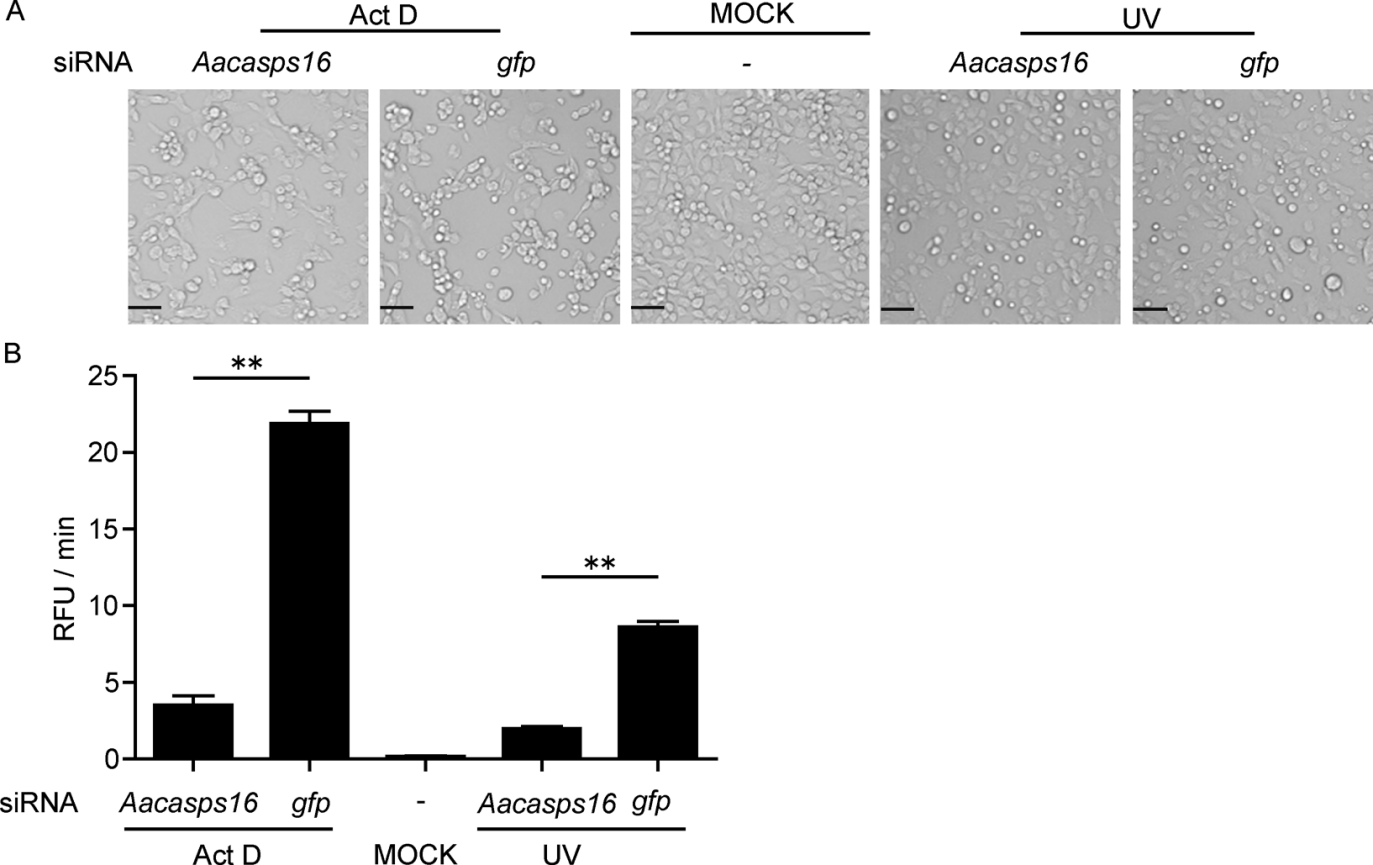
Silencing of *Aacasps16* led to decreased apoptosis induced by UV and Act D treatment. 5×10^5^ C6/36 cells were transfected with siRNA (200 pmol) of *Aacasps16*-siRNA or *gfp*-siRNA, and 24 h later, the cells were treated with 200 μJ/cm^2^ UV or Act D at a final concentration of 1.0 μg/ml, and at 24 h after treatment, cells were subjected to the following analyses: **(A)** Cell images were taken under a microscope (Scale bar indicated 50 μm). **(B)** Cell lysates were incubated with Ac-DEVD-AFC and subjected to caspase activity assay. Caspase activity was indicated as the changes in relative fluorescence units (RFU) per minute. The data were presented with the SD from three independent experiments and statistical significance was calculated by *t* test, **P< 0.01.

To confirm the role of AaCASPS16 in apoptosis in C6/36 cells, effects of AaCASPS16 silencing on Act D- or UV-induced apoptosis were tested. To that purpose, C6/36 cells were transfected with *Aacasps16*-siRNA for 24 h, which led to a partial knock down of *Aacasps16*, as determined by qRT-PCR and immunoblotting analysis (S1A and S1B Fig), and then treated with Act D or UV (C6/36 cells transfected with *gfp*-siRNA were used as a control). At 24 h after treatments, Act D- or UV- induced apoptosis was partially inhibited by the *Aacasps16* knock down, indicated by a smaller number of apoptotic bodies in *Aacasps16*-siRNA-tranfected C6/36 cells than that in the control *gfp*-siRNA-transfected C6/36 cells (Fig 9A). Consistent with this, silencing of *Aacasps16* decreased caspase activity triggered by Act D and UV treatment in C6/36 cells (Fig 9B). These data is consistent with the idea that AaCASPS16 was involved in Act D- and UV- induced apoptosis in C6/36 cells.

## Discussion

*Aedes albopictus*, an important vector for the transmission of arboviruses, is becoming a significant threat to public health due to its fast adaption to new locations. Understanding antiviral defense mechanisms in *Aedes albopictus* would help to develop strategies to control arbovirus transmission. Apoptosis has been reported to be involved in antiviral immunity in insects, while there is less study on apoptosis in *Aedes albopictus*. This study identified a homolog of *Drosophila strica*, *Aacasps16*, as an important apoptotic caspase in *Aedes albopictus* C6/36 cells.

Synthetic caspase substrates are widely used in detecting caspase activity and distinguishing between initiator caspases and effector caspases. Although AaCASPS16 doesn’t contain a long prodomain, purified AaCASPS16 exhibited the strongest activity on initiator caspase substrates and exhibited weak activity on effector caspase substrates, indicating that AaCASPS16 is a potential initiator caspase.

Our study identified AaCASPS16 as a pro-apoptosis caspase: (1) Over-expression of AaCASPS16 triggered apoptosis in C6/36 cells; (2) The processing of AaCASPS16, a hallmark of activation of caspase, was observed in apoptosis triggered by UV and Act D treatment; and (3) Apoptosis induced by UV or Act D treatment was attenuated when *Aacasps16* was partially silenced in C6/36 cells. Strica was reported as a functionally redundant caspase with Dronc in apoptosis. Thus, it is an interesting point for further investigation on whether AaCASPS16 functions redundantly with Dronc homolog in *Aedes albopictus* in the same manner.

Apoptosis was induced by transient transfection of the plasmid pIE1-CASPS16-Flag into C6/36 cells. Immunoblotting with antibody against Flag-tag exhibited an increased amount of protein corresponding to the full-length band of AaCASPS16 when apoptosis was inhibited by adding the caspase inhibitor z-VAD-FMK, however, we have been unable to detect processing band of AaCASPS16 until proteasome inhibitor MG132 was added. Likewise, the endogenous processing band of AaCASPS16, produced during the Act D-triggered apoptosis, was significantly increased with MG132 treatment, indicating that the processed AaCASPS16 was unstable and underwent degradation during apoptosis (S2 Fig). Similar cases were also reported as a kind of protection to the cell from excessive apoptosis by eliminating the cleaved active forms [30, 31].

According to the “induced proximity model”, initiator caspases rely on a recruitment domain (e.g., CARD or DED) in the N-terminal prodomain for dimerization, followed by cleavage and self-activation. AaCASPS16 bears a highly serine- and threonine-rich prodomain with no homology to other previously characterized motifs. An important question in this regard relates to the regulatory role of the activation of AaCASPS16. Using the available database, 49 potential phosphorylation sites existed in the 117-animo acid-prodomain and several potential kinases were predicted. So it remains to be elucidated whether the activation of AaCASPS16 is regulated by phosphorylation.

Given that Strica is insect-specific and has not been extensively studied, studies on the roles of Strica homologs in mosquitoes are extremely important and will help us achieve a better understanding of apoptosis in insects.

## Supporting Information

S1 Fig Effects of silencing of *Aacasps16* at mRNA and protein level. 5×10^5^ C6/36 cells were transfected with siRNA (200 pmol) of *Aacasps16*-siRNA or *gfp*-siRNA, and 24 h later, cells were harvested and subjected to the following analysis: **(A)** Total RNAs were prepared from C6/36 cells and subjected to qRT-PCR analysis. **(B)** Cell lysates were subjected to immunoblotting using antibody against AaCASPS16 and β-actin.
(TIF)

S2 Fig The processed AaCASPS16 was unstable in apoptosis triggered by Act D treatment. C6/36 cells were treated with Act D (1.0 μg/ml) for 24 h. MG132 was added at 8 h before the cells were harvested. Cell lysates were prepared and subjected to Western blotting using an antibody against AaCASPS16. “*” indicated the non-specific band which was regarded as the loading control. A short vertical black line was used to indicate where lanes were removed and separate parts of the same Western blot image were joined together.
(TIF)

S1 Table Gene information used for alignment and phylogenetic analyses. (DOCX)

S2 Table The siRNA sense sequences targeting *Aacasps16* and *gfp*. (DOCX)

S3 Table Primers sequences for qRT-PCR. (DOCX)
