# Supplementary figures and images for: The Strica Homolog AaCASPS16 Is Involved in Apoptosis in the Yellow Fever Vector, Aedes albopictus
Source: PLoS One. 2016 Jun 28;11(6):e0157846. doi: 10.1371/journal.pone.0157846 (PMC4924790; doi:10.1371/journal.pone.0157846)

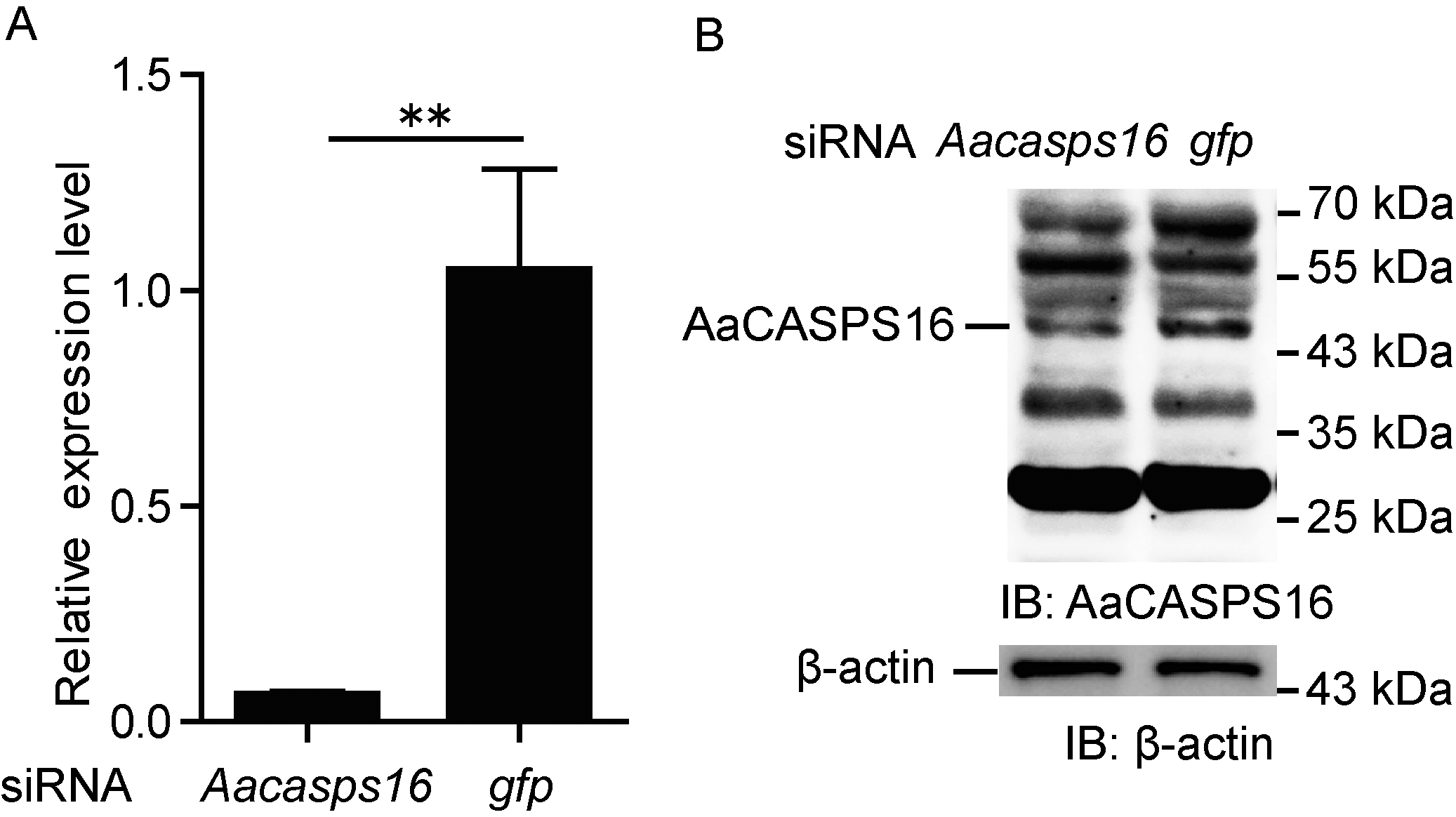

Supplement: S1 Fig — 5×10^5^ C6/36 cells were transfected with siRNA (200 pmol) of Aacasps16-siRNA or gfp-siRNA, and 24 h later, cells were harvested and subjected to the following analysis: (A) Total RNAs were prepared from C6/36 cells and subjected to qRT-PCR analysis. (B) Cell lysates were subjected to immunoblotting using antibody against AaCASPS16 and β-actin. (TIF) [file pone.0157846.s001.tif]

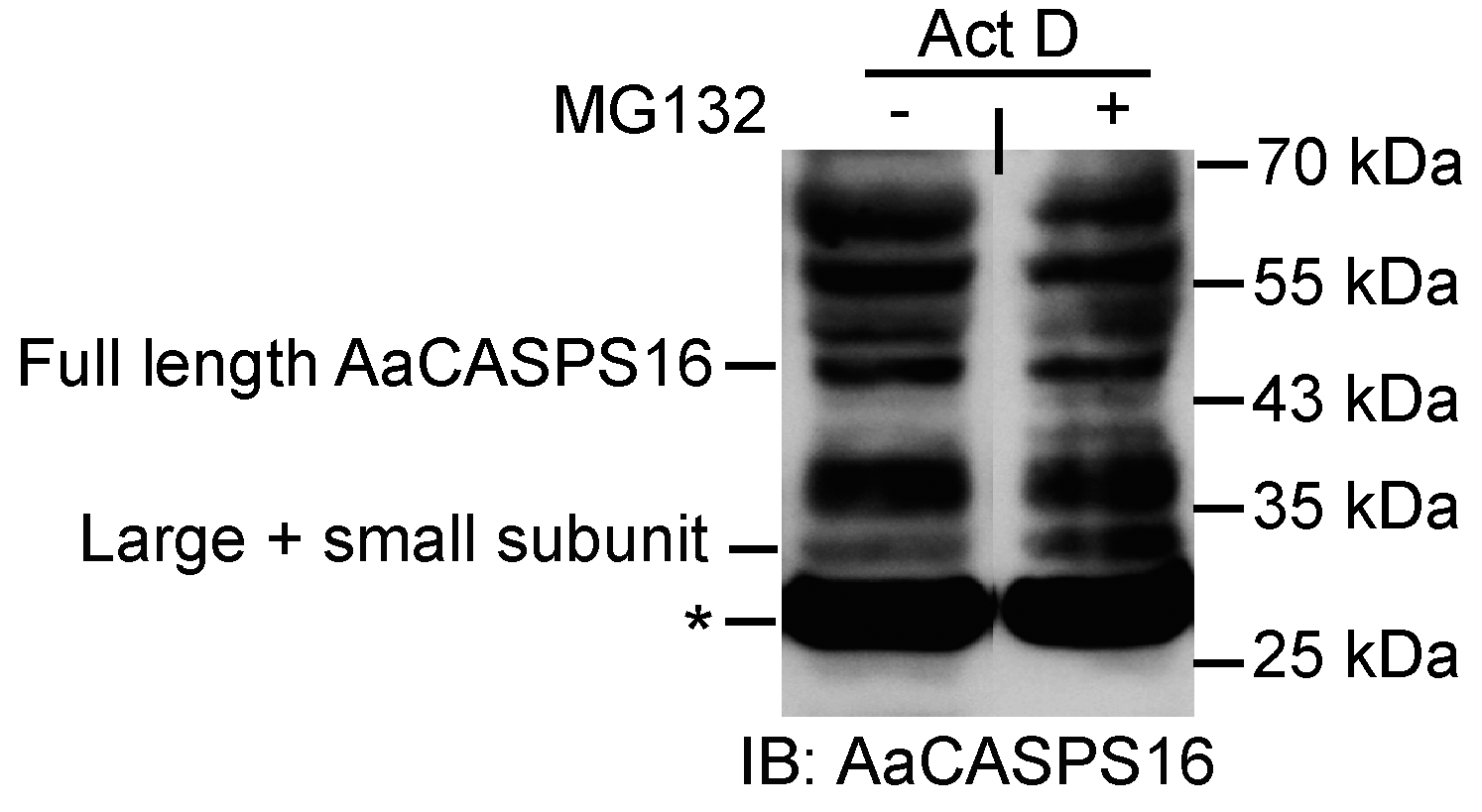

Supplement: S2 Fig — C6/36 cells were treated with Act D (1.0 μg/ml) for 24 h. MG132 was added at 8 h before the cells were harvested. Cell lysates were prepared and subjected to Western blotting using an antibody against AaCASPS16. “*” indicated the non-specific band which was regarded as the loading control. A short vertical black line was used to indicate where lanes were removed and separate parts of the same Western blot image were joined together. (TIF) [file pone.0157846.s002.tif]
