# Supplementary material for: The Strica Homolog AaCASPS16 Is Involved in Apoptosis in the Yellow Fever Vector, Aedes albopictus
Source: PLoS One. 2016 Jun 28;11(6):e0157846. doi: 10.1371/journal.pone.0157846 (PMC4924790; doi:10.1371/journal.pone.0157846)
Supplement: S1 Table — (DOCX) [file pone.0157846.s003.docx]

**S1 Table. Gene information used for alignment and phylogenetic analysis.**

| gene | species | accession numbers | |
| --- | --- | --- | --- |
|  |  | GenBank | VectorBase |
| *dronc* | *Drosophila* | AAF50180 |  |
| *dredd* | *Drosophila* | EDX00805 |  |
| *strica* | *Drosophila* | AAF78902 |  |
| *damm* | *Drosophila* | EDW90874 |  |
| *drice* | *Drosophila* | AAF56939 |  |
| *decay* | *Drosophila* | AAD54071 |  |
| *dcp-1* | *Drosophila* | AAF47027 |  |
| *Aecasps15* | *Aedes aegypti* | EAT42503 | AAEL005963 |
| *Aecasps16* | *Aedes aegypti* | EAT42502 | AAEL005956 |
| *Aecasps17* | *Aedes aegypti* | EAT42501 | AAEL005955 |
| *Aecasps21* | *Aedes aegypti* | EJY57615 | AAEL017498 |
| *Cqcasps27* | *Culex quinquefasciatus* | EDS37507 | CPIJ012579 |
| *Cqcasps28* | *Culex quinquefasciatus* | EDS37508 | CPIJ012580 |
| *Agcasps9* | *Anopheles gambiae* | EAA05460 | AGAP010828 |
| *Agcasps10* | *Anopheles gambiae* | EAA05494 | AGAP010827 |
| *Agcasps12* | *Anopheles gambiae* | EAA05458 | AGAP010829 |
| *Agcasps13* | *Anopheles gambiae* | EAA05456 | AGAP010826 |
