# Supplementary material for: The Strica Homolog AaCASPS16 Is Involved in Apoptosis in the Yellow Fever Vector, Aedes albopictus
Source: PLoS One. 2016 Jun 28;11(6):e0157846. doi: 10.1371/journal.pone.0157846 (PMC4924790; doi:10.1371/journal.pone.0157846)
Supplement: S2 Table — (DOCX) [file pone.0157846.s004.docx]

**S2 Table. The siRNA sense sequences targeting *Aacasps16* and *gfp.***

| siRNA | sense strand sequence(5'-3') | position from starting codon |
| --- | --- | --- |
| *Aacasps16* | AGCCAAAATGCTCTATCAAAAGC | 289-311 |
| *gfp* | CGGCAAGCTGACCCTGAAGTTCA | 120-142 |
