# Supplementary material for: The Strica Homolog AaCASPS16 Is Involved in Apoptosis in the Yellow Fever Vector, Aedes albopictus
Source: PLoS One. 2016 Jun 28;11(6):e0157846. doi: 10.1371/journal.pone.0157846 (PMC4924790; doi:10.1371/journal.pone.0157846)
Supplement: S3 Table — (DOCX) [file pone.0157846.s005.docx]

**S3 Table. Primers sequences for qRT-PCR**

| primers | sequence (5'-3') | position from starting codon |
| --- | --- | --- |
| *Aacasps16* forward | AACCACTAAGATTGTTTCCAAA | 66-87 |
| *Aacasps16* reverse | CCTCGAAGGGAACTATACGCTG | 248-269 |
| *s7* forward | GTCCACGATCCCGCACTCT | 341-359 |
| *s7* reverse | GTGGTCTGCTGGTTCTTGTCC | 468-488 |
